# Supplementary material for: Bayesian multilevel multivariate logistic regression for superiority decision-making under observable treatment heterogeneity
Source: BMC Med Res Methodol. 2023 Oct 5;23:220. doi: 10.1186/s12874-023-02034-z (PMC10552398; doi:10.1186/s12874-023-02034-z)
Supplement: Supplementary file 1 — Additional file 1. [file 12874_2023_2034_MOESM1_ESM.pdf]

Appendix A: Gibbs sampling procedure based on Pólya-Gamma expansion

A.1 Random effects model

Bayesian analysis relies on the posterior distribution of regression coefficients, which is proportional to the likelihood of the data and the prior distribution:

$$p(\boldsymbol{\gamma}_j^q, \boldsymbol{\gamma}^q, \boldsymbol{\Sigma}^q|\boldsymbol{y}) \propto p(\boldsymbol{y}|\boldsymbol{\gamma}_j^q)p(\boldsymbol{\gamma}_j^q|\boldsymbol{\gamma}^q, \boldsymbol{\Sigma}^q)p(\boldsymbol{\gamma}^q)p(\boldsymbol{\Sigma}^q). \tag{1}$$

The multinomial logistic likelihood (Equation 2) can be expanded with a Pólya-Gamma auxiliary variable to suit a Gibbs sampling procedure. This expansion relies on the following equality [1]:

$$\begin{aligned} p((\boldsymbol{y}_j = \boldsymbol{h}^q)|\boldsymbol{\gamma}_j^q, \boldsymbol{\gamma}_j^{-q}, \boldsymbol{\omega}_j^q &= \frac{\exp(\boldsymbol{x}_{ji}\boldsymbol{\gamma}_j^q)}{\sum_{r=1}^{Q-1}\exp(\boldsymbol{x}_{ji}\boldsymbol{\gamma}_j^r) + 1}, \\ &\propto \exp\left[-\frac{1}{2}(\boldsymbol{\kappa}_j^q - \boldsymbol{\eta}_j^q)^T\boldsymbol{\Omega}_j^q(\boldsymbol{\kappa}_j^q - \boldsymbol{\eta}_j^q)\right], \end{aligned} \tag{2}$$

where $\boldsymbol{X}_j$ is a matrix filled with n_j rows of covariate vectors $\boldsymbol{x}_{ji}$ and $\boldsymbol{\eta}_j^q = \boldsymbol{X}_j\boldsymbol{\gamma}_j^q - \ln[\sum_{m\neq q}\exp(\boldsymbol{X}_j\boldsymbol{\gamma}_j^m)]$, $\boldsymbol{\kappa}_j^q = \frac{I(\boldsymbol{y}_j=\boldsymbol{h}^q)-\frac{1}{2}}{\boldsymbol{\omega}_j^q}$.

Equation 2 can be recognized as the kernel of a multivariate Gaussian likelihood of working variable $\boldsymbol{\kappa}_j^q$ [1]:

$$\boldsymbol{\kappa}_j^q \sim N\left(\boldsymbol{\eta}_j^q, \{\boldsymbol{\Omega}_j^q\}^{-1}\right) \tag{3}$$

Here, $\boldsymbol{\Omega}_j^q$ reflects the diagonal matrix of Pólya-Gamma distributed variables $\boldsymbol{\omega}_j^q = (\omega_{j1}^q, \ldots, \omega_{jn_j}^q)$. A Gibbs sampler can be constructed when the likelihood in Equation 3 is combined with multivariate normal prior distributions on random regression coefficients $\boldsymbol{\gamma}_j^q|\boldsymbol{\gamma}^q, \boldsymbol{\Sigma}^q$ and mean random regression coefficients $\boldsymbol{\gamma}^q$, and an inverse-Wishart prior distribution on covariance matrix $\boldsymbol{\Sigma}^q$:

$$\begin{aligned} \boldsymbol{\gamma}_j^q &\sim N(\boldsymbol{\gamma}^q, \boldsymbol{\Sigma}^q) \\ \boldsymbol{\gamma}^q &\sim N(\boldsymbol{g}^q, \boldsymbol{G}^q) \\ \boldsymbol{\Sigma}^q &\sim \mathcal{W}^{-1}(j^0, \boldsymbol{S}^q) \end{aligned} \tag{4}$$

The resulting Gibbs sampler consists of the following steps:

1 Sample mean regression coefficients:

$$\boldsymbol{\gamma}^{q(l)} \sim N\left(\boldsymbol{V}_{\boldsymbol{\gamma}}^q(\{\boldsymbol{\Sigma}^{q(l-1)}\}^{-1}\sum_{j=1}^{J}\boldsymbol{\gamma}_j^{q(l-1)} + \boldsymbol{G}^q\boldsymbol{g}^q), \boldsymbol{V}_{\boldsymbol{\gamma}}^q\right)$$

with prior mean vector $\boldsymbol{g}^q$, prior precision matrix $\boldsymbol{G}^q$ and posterior variance matrix $\boldsymbol{V}_{\boldsymbol{\gamma}} = (J\{\boldsymbol{\Sigma}^{q(l-1)}\}^{-1} + \boldsymbol{G}^q)^{-1}$.

2 Sample covariance matrices of regression coefficients:

$$\boldsymbol{\Sigma}^{q(l)} \sim \mathcal{W}^{-1}\left(j^0 + J, \boldsymbol{S}^q + \sum_{j=1}^{J}\left(\boldsymbol{\gamma}_j^{q(l)} - \boldsymbol{\gamma}^{q(l-1)}\right)\left(\boldsymbol{\gamma}_j^{q(l)} - \boldsymbol{\gamma}^{q(l-1)}\right)^T\right)$$

with prior hyperparameters $j^0 \geq P$ and $\boldsymbol{S}^q$.

3 For each j, sample random regression coefficients:

$$\boldsymbol{\gamma}_j^{q(l)} \sim N\Bigg(\boldsymbol{V}_{\boldsymbol{\gamma}_j^q}^q \big(\boldsymbol{X}_j \boldsymbol{\Omega}_j^{q(l-1)}(\boldsymbol{\kappa}_j^{q(l-1)} + \ln[\sum_{m \neq q} \exp(\boldsymbol{X}_j \boldsymbol{\gamma}_j^{m(l)})]) + \{\boldsymbol{\Sigma}^{q(l)}\}^{-1}\boldsymbol{\gamma}^{q(l)}\big), \boldsymbol{V}_{\boldsymbol{\gamma}_j}^q\Bigg)$$

with prior mean vector $\boldsymbol{\gamma}^{q(l)}$, prior precision matrix $\boldsymbol{\Sigma}^{q(l)}$, posterior variance matrix $\boldsymbol{V}_{\boldsymbol{\gamma}_j}^q = (\boldsymbol{X}_j{}^T \boldsymbol{\Omega}_j^{q(l-1)} \boldsymbol{X}_j + \{\boldsymbol{\Sigma}^{q(l)}\}^{-1})^{-1}$, and diagonal matrix of Pólya-Gamma variables $\boldsymbol{\Omega}_j^{q(l-1)} = \text{diag}(\omega_{j1}^{q(l-1)}, \ldots, \omega_{jn_j}^{q(l-1)})$.

4 For each j and i, sample Pólya-Gamma variables:

$$\omega_{ji}^{q(l)} \sim PG(1, \eta_{ji}^{q(l)})$$

The remainder of this section shows the derivations of the full conditional distributions.

A.1.1 Deriving the likelihood function

The following equality forms the basis to rewrite the multinomial likelihood in Equation 2 as a Gaussian likelihood [1]:

$$\begin{aligned} p((\boldsymbol{y}_j = \boldsymbol{h}^q)|\gamma_j, \boldsymbol{\omega}_j^q, \boldsymbol{x}_j) &= \frac{\exp(\boldsymbol{x}_{ji}\boldsymbol{\gamma}_j^q)}{\sum_{r=1}^{Q-1} \exp(\boldsymbol{x}_{ji}\boldsymbol{\gamma}_j^r) + 1}, \\ &= \prod_{i=1}^{n_j} 2 \exp\left[\kappa_{ji}^q \omega_{ji}^q \eta_{ji}^q\right] \int_0^\infty \exp\left[\frac{-\omega_{ji}^q (\eta_{ji}^q)^2}{2}\right] p(\omega_{ji}^q) d\omega_{ji}^q \end{aligned} \quad (5)$$

where $\omega_{ji}^q \sim PG(1, \eta_{ji}^q)$ is a Pólya-Gamma distributed variable,
where $\eta_{ji}^q = \boldsymbol{x}_{ji}\boldsymbol{\gamma}_j^q - \ln\left[\sum_{m \neq q} \exp(\boldsymbol{x}_{ji}\boldsymbol{\gamma}_j^m)\right]$,
and where working variable $\boldsymbol{\kappa}_j^q = \frac{I(\boldsymbol{y}_j = \boldsymbol{h}^q) - \frac{1}{2}}{\boldsymbol{\omega}_j^q}$.

Further algebraic transformation results in the kernel of a Gaussian likelihood:

$$
\begin{aligned}
p((\boldsymbol{y}_j = \boldsymbol{h}^q)|.) &= \prod_{i=1}^{n_j} 2 \exp\left[\kappa_{ji}^q \omega_{ji}^q \eta_{ji}^q\right] \int_0^\infty \exp\left[\frac{-\omega_{ji}^q (\eta_{ji}^q)^2}{2}\right] p(\omega_{ji}^q) d\omega_{ji}^q \\
&\propto \exp\left[\frac{1}{2}(\boldsymbol{\kappa}_j^q \boldsymbol{\omega}_j^q \boldsymbol{\eta}_j^q - \boldsymbol{\omega}_j^q (\boldsymbol{\eta}_j^q)^2\right] \\
&\propto \exp\left[-\frac{1}{2}\left(\boldsymbol{\kappa}_j^q - \boldsymbol{\eta}_j^q\right)^T \boldsymbol{\Omega}_j^q \left(\boldsymbol{\kappa}_j^q - \boldsymbol{\eta}_j^q\right)\right],
\end{aligned}
\tag{6}
$$

Hence, working variable $\boldsymbol{\kappa}_j^q$ is multivariate normally distributed:

$$
\boldsymbol{\kappa}_j^q \sim N\left(\boldsymbol{\eta}_j^q, \{\boldsymbol{\Omega}_j^q\}^{-1}\right). \tag{7}
$$

A.1.2 Deriving conditional posterior distributions

Random regression coefficients $\boldsymbol{\gamma}_j^q$ Using the likelihood in Equation 7 and prior distribution $\boldsymbol{\gamma}_j^q \sim N(\boldsymbol{\gamma}^q, \{\boldsymbol{\Sigma}^q\})$, the conditional posterior distribution of random regression coefficients $\boldsymbol{\gamma}_j^q$ is also a multivariate normal distribution:

$$
\begin{aligned}
p(\boldsymbol{\gamma}_j^q|.) \propto & p(\boldsymbol{y}_j|\boldsymbol{\gamma}_j^q, \boldsymbol{\gamma}_j^{-q}, \boldsymbol{\omega}_j^q, \boldsymbol{x}) p(\boldsymbol{\gamma}_j^q) \\
\propto & \exp\left[-\frac{1}{2}\left(\boldsymbol{\kappa}_j^q - (\boldsymbol{\eta}_j^q)\right)^T \boldsymbol{\Omega}_j^q \left(\boldsymbol{\kappa}_j^q - (\boldsymbol{\eta}_j^q)\right)\right] \times \\
& \exp\left[-\frac{1}{2}\left(\boldsymbol{\gamma}_j^q - \boldsymbol{\gamma}^q\right)^T \{\boldsymbol{\Sigma}^q\}^{-1} \left(\boldsymbol{\gamma}_j^q - \boldsymbol{\gamma}^q\right)\right] \\
\propto & \exp\Bigg[-\frac{1}{2}\Big(\{\boldsymbol{\gamma}_j^q\}^T(\{\boldsymbol{X}_j\}^T \boldsymbol{\Omega}_j^q \boldsymbol{X}_j + \{\boldsymbol{\Sigma}^q\}^{-1})\boldsymbol{\gamma}_j^q - 2\{\boldsymbol{\gamma}_j^q\}^T \\
& \quad (\{\boldsymbol{X}_j\}^T \boldsymbol{\Omega}_j^q (\boldsymbol{\kappa}_j^q + \ln[\sum_{m \neq q} \exp(\boldsymbol{X}_j \boldsymbol{\gamma}_j^m)]) + \{\boldsymbol{\Sigma}^q\}^{-1}\boldsymbol{\gamma}^q)\Big)\Bigg] \\
\propto & \exp\Bigg[-\frac{1}{2} \\
& \left(\boldsymbol{\gamma}_j^q - \boldsymbol{V}_{\gamma_j}^q(\{\boldsymbol{X}_j\}^T \boldsymbol{\Omega}_j^q (\boldsymbol{\kappa}_j^q + \ln[\sum_{m \neq q} \exp(\boldsymbol{X}_j \boldsymbol{\gamma}_j^m)]) + \{\boldsymbol{\Sigma}^q\}^{-1}\boldsymbol{\gamma}^q)\right)^T \\
& \{\boldsymbol{V}_{\gamma_j}^q\}^{-1} \\
& \left(\boldsymbol{\gamma}_j^q - \boldsymbol{V}_{\gamma_j}^q(\{\boldsymbol{X}_j\}^T \boldsymbol{\Omega}_j^q (\boldsymbol{\kappa}_j^q + \ln[\sum_{m \neq q} \exp(\boldsymbol{X}_j \boldsymbol{\gamma}_j^m)]) + \{\boldsymbol{\Sigma}^q\}^{-1}\boldsymbol{\gamma}^q)\right)\Bigg] \\
\sim & N\left(\boldsymbol{V}_{\gamma_j}^q(\boldsymbol{X}_j \boldsymbol{\Omega}_j^q (\boldsymbol{\kappa}_j^q + \ln[\sum_{m \neq q} \exp(\boldsymbol{X}_j \boldsymbol{\gamma}_j^m)]) + \{\boldsymbol{\Sigma}^q\}^{-1}\boldsymbol{\gamma}^q), \boldsymbol{V}_{\gamma_j}^q\right)
\end{aligned}
\tag{8}
$$

with prior mean vector $\boldsymbol{\gamma}^q$, prior variance matrix $\boldsymbol{\Sigma}^q$ and posterior variance matrix $\boldsymbol{V}_{\gamma_j}^q = (\boldsymbol{X}_j{}^T \boldsymbol{\Omega}_j^q \boldsymbol{X}_j + \{\boldsymbol{\Sigma}^q\}^{-1})^{-1}$.

Random mean $\boldsymbol{\gamma}^q$ When the posterior distribution of $\boldsymbol{\gamma}_j^q$ (Equation 8) is included as a likelihood and combined with a $N(\boldsymbol{g}^q, \{\boldsymbol{G}^q\}^{-1})$ prior distribution, the conditional posterior distribution of random mean $\boldsymbol{\gamma}^q$ is another multivariate normal distribution:

$$\begin{aligned}
p(\boldsymbol{\gamma}^q|.) \propto & \prod_{j=1}^{J} p(\boldsymbol{\gamma}_j^q|\boldsymbol{\gamma}^q, \boldsymbol{\Sigma}^q)p(\boldsymbol{\gamma}^q) \qquad (9)\\
\propto & \prod_{j=1}^{J} \exp\left[-\frac{1}{2}(\boldsymbol{\gamma}_j^q - \boldsymbol{\gamma}^q)^T\{\boldsymbol{\Sigma}^q\}^{-1}(\boldsymbol{\gamma}_j^q - \boldsymbol{\gamma}^q)\right] \times \\
& \exp\left[-\frac{1}{2}(\boldsymbol{\gamma}^q - \boldsymbol{g}^q)^T\boldsymbol{G}^q(\boldsymbol{\gamma}^q - \boldsymbol{g}^q)\right] \\
\propto & \exp\left[-\frac{1}{2}(\{\boldsymbol{\gamma}^q\}^T\left(J\{\boldsymbol{\Sigma}^q\}^{-1}\right)\boldsymbol{\gamma}^q) - 2\{\boldsymbol{\gamma}^q\}^T\left(\{\boldsymbol{\Sigma}^q\}^{-1}\sum_{j=1}^{J}\boldsymbol{\gamma}_j^q\right)\right] \times \\
& \exp\left[-\frac{1}{2}\{\boldsymbol{\gamma}^q\}^T\boldsymbol{G}^q\boldsymbol{\gamma}^q - 2\{\boldsymbol{\gamma}^q\}^T\boldsymbol{G}^q\boldsymbol{g}^q\right] \\
\propto & \exp\left[-\frac{1}{2}\{\boldsymbol{\gamma}^q\}^T\left(J\{\boldsymbol{\Sigma}^q\}^{-1} + \boldsymbol{G}^q\right)\boldsymbol{\gamma}^q - \right. \\
& \left. 2\{\boldsymbol{\gamma}^q\}^T\left(\{\boldsymbol{\Sigma}^q\}^{-1}\sum_{j=1}^{J}\boldsymbol{\gamma}_j^q + \boldsymbol{G}^q\boldsymbol{g}^q\right)\right] \\
\propto & \exp\left[-\frac{1}{2}\left(\boldsymbol{\gamma}^q - \boldsymbol{V}_{\boldsymbol{\gamma}}^q\left(\{\boldsymbol{\Sigma}^q\}^{-1}\sum_{j=1}^{J}\boldsymbol{\gamma}_j^q + \boldsymbol{G}^q\boldsymbol{g}^q\right)\right)^T\{\boldsymbol{V}_{\boldsymbol{\gamma}}^q\}^{-1}\right. \\
& \left.\left(\boldsymbol{\gamma}^q - \boldsymbol{V}_{\boldsymbol{\gamma}}^q\left(\{\boldsymbol{\Sigma}^q\}^{-1}\sum_{j=1}^{J}\boldsymbol{\gamma}_j^q + \boldsymbol{G}^q\boldsymbol{g}^q\right)\right)\right] \\
\sim & N\left(\boldsymbol{V}_{\boldsymbol{\gamma}}^q\left(\{\boldsymbol{\Sigma}^q\}^{-1}\sum_{j=1}^{J}\boldsymbol{\gamma}_j^q + \boldsymbol{G}^q\boldsymbol{g}^q\right), \boldsymbol{V}_{\boldsymbol{\gamma}}^q\right),
\end{aligned}$$

with prior mean vector $\boldsymbol{g}^q$, prior precision matrix $\boldsymbol{G}^q$, and posterior variance matrix $\boldsymbol{V}_{\boldsymbol{\gamma}} = (J\{\boldsymbol{\Sigma}^q\}^{-1} + \boldsymbol{G}^q)^{-1}$.

Random variance $\boldsymbol{\Sigma}^q$ When the posterior distribution of $\boldsymbol{\gamma}_j^q$ (Equation 8) is included as a likelihood and combined with an inverse Wishart $\mathcal{W}^{-1}(j^0, \boldsymbol{S}^q)$ prior, the conditional posterior distribution of random variance $\boldsymbol{\Sigma}^q$ is proportional to an

inverse Wishart distribution:

$$
\begin{aligned}
p(\boldsymbol{\Sigma}^q|.) &\propto p(\boldsymbol{\gamma}_j^q|\boldsymbol{\gamma}^q, \boldsymbol{\Sigma}^q)p\{\boldsymbol{\Sigma}^q\} \\
&\propto \prod_{j=1}^{J} |\boldsymbol{\Sigma}^q|^{\frac{1}{2}} \exp\left[-\frac{1}{2}(\boldsymbol{\gamma}_j^q - \boldsymbol{\gamma}^q)^T\{\boldsymbol{\Sigma}^q\}^{-1}(\boldsymbol{\gamma}_j^q - \boldsymbol{\gamma}^q)\right] \times \\
&\qquad |\boldsymbol{\Sigma}^q|^{\frac{1}{2}(j^0+p^R+1)} \exp\left[-\frac{1}{2}tr(\boldsymbol{S}^q\{\boldsymbol{\Sigma}^q\}^{-1})\right] \\
&\propto |\boldsymbol{\Sigma}^q|^{-\frac{1}{2}(j^0+J+P^R+1)} \times \\
&\qquad \exp\left[-\frac{1}{2}tr\left(\left(\boldsymbol{S}^q + \sum_{j=1}^{J}(\boldsymbol{\gamma}_j^q - \boldsymbol{\gamma}^q)(\boldsymbol{\gamma}_j^q - \boldsymbol{\gamma}^q)^T\right)\{\boldsymbol{\Sigma}^q\}^{-1}\right)\right] \\
&\sim \mathcal{W}^{-1}\left(j^0 + J, \boldsymbol{S}^q + \sum_{j=1}^{J}(\boldsymbol{\gamma}_j^q - \boldsymbol{\gamma}^q)(\boldsymbol{\gamma}_j^q - \boldsymbol{\gamma}^q)^T\right).
\end{aligned}
\tag{10}
$$

A.2 Mixed effects model

A mixed effect model is defined as follows:

$$
\boldsymbol{\phi}_{ji}^q = f(\boldsymbol{x}_{ji}^F\boldsymbol{\beta}^q + \boldsymbol{x}_{ji}^R\boldsymbol{\gamma}_j^q) \tag{11}
$$

where $\boldsymbol{x}_{ji}^F$ and $\boldsymbol{x}_{ji}^R$ are vectors of fixed and random covariates respectively. Vectors $\boldsymbol{\beta}^q$ and $\boldsymbol{\gamma}_j^q$ reflect the accompanying fixed and random regression coefficients. Function f refers to the multinomial logistic likelihood function.

The multivariate normal distribution of working variable $\boldsymbol{\kappa}_j^q$ then has the following form:

$$
\boldsymbol{\kappa}_j^q \sim N\left(\boldsymbol{\eta}_j^q, \{\boldsymbol{\Omega}_j^q\}^{-1}\right). \tag{12}
$$

Here, $\boldsymbol{\eta}_j^q = \boldsymbol{X}_j^F\boldsymbol{\beta}^q + \boldsymbol{X}_j^R\boldsymbol{\gamma}_j^q - \ln[\sum_{m\neq q}\exp(\boldsymbol{X}_j^F\boldsymbol{\beta}^m + \boldsymbol{X}_j^R\boldsymbol{\gamma}_j^m)]$. The likelihood in Equation 12 can be combined with the prior distributions in Equation 4, complemented with a multivariate normally distributed prior on $\boldsymbol{\beta}^q$:

$$
\boldsymbol{\beta}^q \sim N(\boldsymbol{b}^q, \boldsymbol{B}^q) \tag{13}
$$

The Gibbs sampling algorithm in list A.1 is extended with a distinct step for the fixed regression coefficients:

1 Sample fixed regression coefficients:

$$
\begin{aligned}
\boldsymbol{\beta}^{q(l)} \sim N \Bigg(&\boldsymbol{V}_{\boldsymbol{\beta}}^q(\sum_{j=1}^{J}{\boldsymbol{X}_j^F}^T\boldsymbol{\Omega}_j^{q(l-1)}(\boldsymbol{\kappa}_j^{q(l-1)} - \boldsymbol{X}_j^R\boldsymbol{\gamma}_j^{q(l)} + \\
&\ln[\sum_{m\neq q}\exp(\boldsymbol{X}_j^F\boldsymbol{\beta}^{m(l)} + \boldsymbol{X}_j^R\boldsymbol{\gamma}_j^{m(l-1)})]) + \mathbf{B}^q\boldsymbol{b}^q), \boldsymbol{V}_{\boldsymbol{\beta}}^q \Bigg)
\end{aligned}
$$

with prior mean vector $\boldsymbol{b}^q$, prior precision matrix $\mathbf{B}^q$ and posterior variance matrix $\boldsymbol{V}^q_{\boldsymbol{\beta}} = (\sum_{j=1}^{J} {\boldsymbol{X}^F_j}^T \boldsymbol{\Omega}^{q(l-1)}_j \boldsymbol{X}^F_j + \mathbf{B}^q)^{-1}$.

2 Sample mean random regression coefficients:

$$\boldsymbol{\gamma}^{q(l)} \sim N\left(\boldsymbol{V}^q_{\boldsymbol{\gamma}}(\{\boldsymbol{\Sigma}^{q(l-1)}\}^{-1}\sum_{j=1}^{J}\boldsymbol{\gamma}^{q(l-1)}_j + \boldsymbol{G}^q\boldsymbol{g}^q), \boldsymbol{V}^q_{\boldsymbol{\gamma}}\right)$$

with prior mean vector $\boldsymbol{g}^q$, prior precision matrix $\boldsymbol{G}^q$ and posterior variance matrix $\boldsymbol{V}_{\boldsymbol{\gamma}} = (J\{\boldsymbol{\Sigma}^{q(l-1)}\}^{-1} + \boldsymbol{G}^q)^{-1}$.

3 Sample covariance matrices of random regression coefficients:

$$\boldsymbol{\Sigma}^{q(l)} \sim \mathcal{W}^{-1}\left(j^0 + J, \boldsymbol{\Sigma}^0 + \sum_{j=1}^{J}\left(\boldsymbol{\gamma}^{q(l-1)}_j - \boldsymbol{\gamma}^{q(l)}\right)\left(\boldsymbol{\gamma}^{q(l-1)}_j - \boldsymbol{\gamma}^{q(l)}\right)^T\right)$$

with prior hyperparameters $j^0 \geq P^R$ and $\boldsymbol{\Sigma}^0$.

4 For each j, sample random regression coefficients:

$$\begin{aligned}\boldsymbol{\gamma}^{q(l)}_j \sim & N\Big(\boldsymbol{V}^q_{\boldsymbol{\gamma}^q_j}(\boldsymbol{X}^R_j\boldsymbol{\Omega}^{q(l-1)}_j(\boldsymbol{\kappa}^{q(l-1)}_j - \boldsymbol{X}^F_j\boldsymbol{\beta}^{q(l)} + \\ & \ln[\sum_{m\neq q}\exp(\boldsymbol{X}^F_j\boldsymbol{\beta}^{m(l)} + \boldsymbol{X}^R_j\boldsymbol{\gamma}^{m(l)}_j)]) + \{\boldsymbol{\Sigma}^q\}^{-1}\boldsymbol{\gamma}^q), \boldsymbol{V}^q_{\boldsymbol{\gamma}_j}\Big)\end{aligned}$$

with prior mean vector $\boldsymbol{\gamma}^{q(l)}$, prior precision matrix $\boldsymbol{\Sigma}^{q(l)}$ and posterior variance matrix $\boldsymbol{V}^q_{\boldsymbol{\gamma}_j} = ({\boldsymbol{X}^R_j}^T\boldsymbol{\Omega}^{q(l-1)}_j\boldsymbol{X}^R_j + \{\boldsymbol{\Sigma}^{q(l)}\}^{-1})^{-1}$.

5 For each j and i, sample Pólya-Gamma variables:

$$\omega^{q(l)}_{ji} \sim PG(1, \eta^{q(l)}_{ji})$$

A.3 A note on prior specification

A.3.1 Regression parameters

In the Gibbs sampling framework, regression coefficients are normally distributed with a mean and covariance matrix. We shortly discuss the role of these parameters below. The covariance matrix defines the spread of the distribution and therefore has a substantial influence on informativity: Small variance parameters increase prior information. When non-informativity is preferable, large variance parameters are not the simple answer, as they may destabilize computations in Bayesian logistic regression analysis [2]. Jeffreys's prior could be an option, but sufficiently stable computation is not guaranteed [2, 3]. The challenge is therefore to specify prior variance parameters that are both sufficiently small to support stable analysis and to give a realistic support of the parameter and at the same time sufficiently large to be considered vague.

The mean hyperparameters defines the center of the distribution and becomes increasingly influential on the posterior distribution when the variance of the distribution is small. The relevance of adequate mean hyperparameters therefore increases with the informativity of the analysis. It should be noted that prior

information of mean regression coefficients is not always available in the required parametrization. Researchers may be more likely to have information available in terms of (success) probabilities rather than logistic regression parameters. Kavelaars et al. propose an approach to compute mean hyperparameters for the context of treatment comparison in the presence of a single patient characteristics, based on expected joint response probabilities [4].

A.3.2 Covariance matrices

The covariance matrix follows an inverse-Wishart distribution with parameters. Specifying a non-informative prior on covariance matrices and variance parameters in general is not straightforward [5, 6]. The informativity of the inverse-Wishart distribution is sensitive to the size of variance parameters: small variances make inverse-Wishart distributions more informative. Naively specifying standard prior hyperparameters without consideration of prior information or data at hand may result in an undesirably large prior influence. Weakly informative (data-based) prior specification may be superior, if not essential for computational stability [5].

Appendix B: Procedure for transformation to the probability scale and decision-making

Algorithm 1 Procedure for statistical decision-making with posterior regression coefficients

Step 1. Transform regression coefficients to treatment differences

Let $\boldsymbol{\gamma}_j^Q = (0, \dots, 0)$ and $\boldsymbol{x} = (1, T, w, \dots)$

for draw $(l) \leftarrow 1 : L$ **do**

  for cluster $j \leftarrow 1 : J$ **do**

    Compute joint response probabilities

    for treatment $T \leftarrow 0 : 1$ **do**

      for joint response category $q \leftarrow 1 : Q$ **do**

        if Population of interest defined by a range of values of w **then**

$$\text{Compute } \phi_{Tj}^{q(l)} = \int_w \frac{\exp\left[\boldsymbol{x}_j' \boldsymbol{\gamma}_j^{q(l)}\right]}{\sum_{r=1}^{Q-1} \exp\left[\boldsymbol{x}_j' \boldsymbol{\gamma}_j^{r(l)}\right] + 1} dw$$

        end if

        if Population of interest defined by a fixed value of w **then**

$$\text{Compute } \phi_{Tj}^{q(l)} = \frac{\exp\left[\boldsymbol{x}_j' \boldsymbol{\gamma}_j^{q(l)}\right]}{\sum_{r=1}^{Q-1} \exp\left[\boldsymbol{x}_j' \boldsymbol{\gamma}_j^{r(l)}\right] + 1}$$

        end if

      end for

      Compute multivariate success probabilities

      for outcome $k \leftarrow 1 : K$ **do**

$$\text{Compute } \theta_{Tj}^{q(l)} = \sum_{q=1}^{Q} \phi_{Tj}^{q(l)} I(\boldsymbol{h}^q \in \boldsymbol{U}_k)$$

        Compute multivariate treatment difference

Compute $\delta_j^{k(l)} = \theta_{1j}^{k(l)} - \theta_{0j}^{k(l)}$
end for
end for
end for
for outcome $k \leftarrow 1 : K$ **do**
Pool $\delta^{k(l)} = \sum_{j=1}^{J} \frac{n_j}{\sum_{j=1}^{J} n_j} \delta_j^{k(l)}$
end for
end for
<u>**Step 2. Make superiority decision**</u>
Define superiority region $\mathcal{S}_R$
Draw conclusion
if $\frac{1}{L} \sum_{(l)=1}^{L} I(\boldsymbol{\delta}^{(l)} \in \mathcal{S}_R) > p_{cut}$ **then** Conclude superiority
else Conclude non-superiority
end if

Author details

References

1. Polson NG, Scott JG, Windle J. Bayesian inference for logistic models using Pólya–Gamma latent variables. Journal of the American statistical Association. 2013;108(504):1339–1349.
2. Gelman A, Jakulin A, Pittau MG, Su YS. A weakly informative default prior distribution for logistic and other regression models. The Annals of Applied Statistics. 2008 dec;2(4):1360–1383.
3. Poirier D. Jeffreys' prior for logit models. Journal of Econometrics. 1994 aug;63(2):327–339.
4. Kavelaars XM, Mulder J, Kaptein MC. Bayesian multivariate logistic regression for superiority and inferiority decision-making under treatment heterogeneity. Submitted for publication. 2022;.
5. Gelman A. Prior distributions for variance parameters in hierarchical models (comment on article by Browne and Draper). Bayesian Analysis. 2006;1(3):515 – 534. Available from: https://doi.org/10.1214/06-BA117A.
6. Schuurman NK, Grasman RPPP, Hamaker EL. A Comparison of Inverse-Wishart Prior Specifications for Covariance Matrices in Multilevel Autoregressive Models. Multivariate Behavioral Research. 2016 mar;51(2-3):185–206.
